# Associations of Dietary Index for Gut Microbiota and Flavonoid Intake With Female Infertility in the United States

**DOI:** 10.1002/fsn3.70098

**Published:** 2025-03-26

**Authors:** Di Xiao, Xiang Sun, Weidong Li, Zihao Wen, Wei‐Hong Zhang, Li Yang

**Affiliations:** ^1^ Department of Comprehensive Maternal and Child Health, Guangzhou Women and Children's Medical Center Guangzhou Medical University Guangzhou China; ^2^ Department of Gynecology and Obstetrics, Guangzhou Women and Children's Medical Center Guangzhou Medical University Guangzhou China; ^3^ International Centre for Reproductive Health (ICRH), Department of Public Health and Primary Care Ghent University Gent Belgium; ^4^ School of Public Health Université Libre de Bruxelles (ULB) Bruxelles Belgium

**Keywords:** age‐stratified analysis, dietary flavonoid intake, DI‐GM, female infertility, healthier gut microbiota diet

## Abstract

This study aimed to investigate the associations between a dietary index for gut microbiota (DI‐GM), flavonoid intake, and female infertility, while exploring age‐specific differences in these relationships to identify potential dietary strategies for female infertility prevention. This cross‐sectional study focused on female participants aged 18–45 years, with data obtained from the 2017–2018 cycle of the National Health and Nutrition Examination Survey (NHANES). Weighted multivariable logistic regression models were employed to examine the associations between DI‐GM, flavonoid intake, and self‐reported female infertility. Age‐stratified analyses were performed to evaluate whether these associations varied across reproductive life stages. Higher DI‐GM scores were significantly associated with reduced infertility risk (aOR = 0.30, 95% CI: 0.13–0.71, *p* = 0.006), with the strongest protective effects observed in women aged < 35 years (Q3: aOR = 0.13, 95% CI: 0.03–0.58, *p* = 0.007; Q4: aOR = 0.27, 95% CI: 0.09–0.77, *p* = 0.015). Beneficial gut microbiota scores also showed a protective effect (aOR = 0.75, 95% CI: 0.57–0.98, *p* = 0.036). Among women aged ≥ 35 years, moderate flavonoid intake (Q2) showed a significant inverse association with female infertility risk (aOR = 0.19, 95% CI: 0.06–0.66, *p* = 0.009). Our findings reveal novel evidence that higher DI‐GM scores and moderate flavonoid intake are significantly linked to a lower risk of female infertility, with age‐specific patterns observed. Higher DI‐GM scores showed significant protective effects in younger women (< 35 years), while moderate flavonoid intake was protective in women aged ≥ 35 years. These findings underscore the potential of personalized dietary strategies targeting gut microbiota composition and flavonoid intake as cost‐effective approaches for female infertility prevention and management across different reproductive life stages.

## Graphical abstract

Higher DI‐GM scores and moderate flavonoid intake showed protective effects against female infertility. DI‐GM benefits were more significant in women under 35, while moderate flavonoid intake was more protective in women aged 35 and above. This study highlights the potential of dietary interventions targeting gut microbiota composition and flavonoid intake, which might be simple, cost‐effective strategies for female infertility prevention and management, with age‐specific applications for women at different stages of reproductive life.

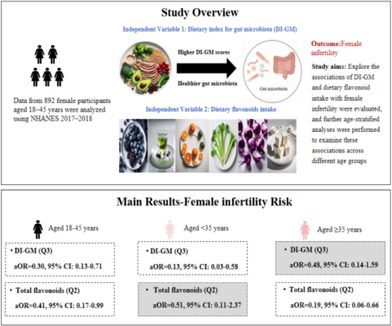

## 1 Introduction

Infertility, characterized as the absence of spontaneous conception within a year after unprotected sexual activity (Practice Committee of the American Society for Reproductive Medicine 2013), is well associated with physical, psychological, emotional, spiritual, and medical challenges in affected individuals (Walker and Tobler 2022). Infertility has been related to psychological distress, social stigmatization, diminished quality of life, and unsatisfactory sexual lives (Janssen et al. 2007). In addition, infertility has demonstrated an association with increased cancer risk (Venn et al. 1995). Infertility is a global epidemic, impacting approximately 15% of couples of reproductive age (Gerrits et al. 2017). In the United States, it has been reported that nearly 13%–16% of females fail to conceive within 1 year or during observed cycles of attempted conception (Gleason et al. 2019). The estimated prevalence of female infertility is 12.5% in the United Kingdom (Datta et al. 2016). In Iran, Vahidi et al. (2009) have found that the prevalence of lifetime primary infertility was 24.9%. Therefore, infertility is a significant global public health issue and a primary challenge in reproductive medicine; however, its pathophysiology remains unclear. Hence, identifying modifiable risk factors remains crucial for the prevention of infertility.

Recent evidence has highlighted the critical role of gut microbiota in female reproductive health (Qi et al. 2021). Dysbiosis of the gut microbiota has been found to be linked to various reproductive disorders that can lead to female infertility, including endometriosis, polycystic ovary syndrome (PCOS), and premature ovarian insufficiency (POI) (Wang, Zheng, et al. 2024). A novel dietary index for gut microbiota (DI‐GM) (Kase et al. 2024) provides the first standardized tool for assessing dietary patterns that influence gut microbiota health. This index effectively recognizes dietary patterns with beneficial or harmful effects on gut microbiota diversity. However, the relationship between this newly proposed DI‐GM and female infertility remains unexplored. Given that gut microbiota dysbiosis has been implicated in various reproductive disorders and that dietary patterns can significantly influence gut microbiota composition (Losno et al. 2021; Qi et al. 2021; Shan et al. 2024), investigating the association between DI‐GM and female infertility is crucial for elucidating the potential mechanisms by which diet‐induced changes in gut microbiota influence fertility outcomes.

In addition to gut microbiota, oxidative stress has emerged as a critical pathogenic factor in female infertility, contributing to various reproductive disorders including poor oocyte quality, implantation failure, and pregnancy complications (Zaha et al. 2023). Recent evidence has demonstrated that natural antioxidants, such as vitamin C, dietary intake can significantly reduce the risk of female infertility (Ji et al. 2023). These findings indicate the potential therapeutic value of dietary antioxidants in fertility treatment. Flavonoids, a class of naturally occurring dietary compounds with potent antioxidant properties, have garnered increasing attention in reproductive health research, as evidenced by the growing number of studies investigating their potential therapeutic applications in fertility treatment (Sirotkin and Harrath 2024). Flavonoids not only exhibit strong antioxidant capacity but also possess anti‐inflammatory properties and can modulate multiple pathways relevant to fertility, including hormone regulation and inflammatory response (Sirotkin and Harrath 2024). While extensive research has established the beneficial effects of dietary flavonoid intake on various health conditions such as cardiovascular disease and metabolic disorders (Blesso 2019), their specific role in female fertility remains unclear. Given the demonstrated benefits of antioxidant supplementation in fertility treatment and the potent antioxidant properties of flavonoids, investigating the association between flavonoid intake and female fertility represents an important research direction.

Despite the emerging evidence linking gut microbiota to reproductive health and the potential benefits of dietary flavonoids as antioxidants in fertility treatment, several critical knowledge gaps remain. Firstly, although the DI‐GM represents a validated approach for evaluating diet‐microbiota relationships, the potential association between DI‐GM and female infertility remains unexplored. Secondly, although flavonoids show promise as dietary antioxidants in reproductive health through their antioxidant and anti‐inflammatory properties, evidence on the relationship between flavonoid intake and female infertility is lacking.

Furthermore, considering that age is a key factor influencing fertility (Chen et al. 2023), female fertility declines significantly with advancing age, with women over 35 years experiencing reduced ovarian reserve, diminished oocyte quality, and an increased risk of infertility and pregnancy complications (Shang et al. 2024). The role of age in fertility outcomes is well established, yet its potential influence on the associations between modifiable risk factors, such as DI‐GM and flavonoid intake, and female infertility risk remains underexplored. Investigating age‐specific patterns in these associations is essential for developing tailored intervention strategies that address the unique female fertility challenges across different life stages.

Therefore, we performed a secondary analysis of 2017–2018 National Health and Nutrition Examination Survey (NHANES) data to examine the associations of DI‐GM and dietary flavonoid intake with female infertility. Additionally, we conducted age‐stratified analyses to examine whether the associations between DI‐GM, flavonoid intake, and female infertility risk vary across different age groups.

## 2 Methods

### 2.1 Data Source and Sample

Data were obtained from the NHANES, a cross‐sectional study conducted continuously and released in 2‐year cycles by the US Centers for Disease Control and Prevention (CDC). A complex, stratified, multistage probability cluster design, including socioeconomic, dietary, physiological, health‐related, and laboratory data, was used to gather NHANES data on civilians randomly selected from the US population. The Ethics Review Board of the National Center for Health Statistics approved the NHANES protocol, and all participants provided informed consent. Moreover, because NHANES data are publicly available, an additional review is not required before obtaining the data for analysis.

**FIGURE 1:**
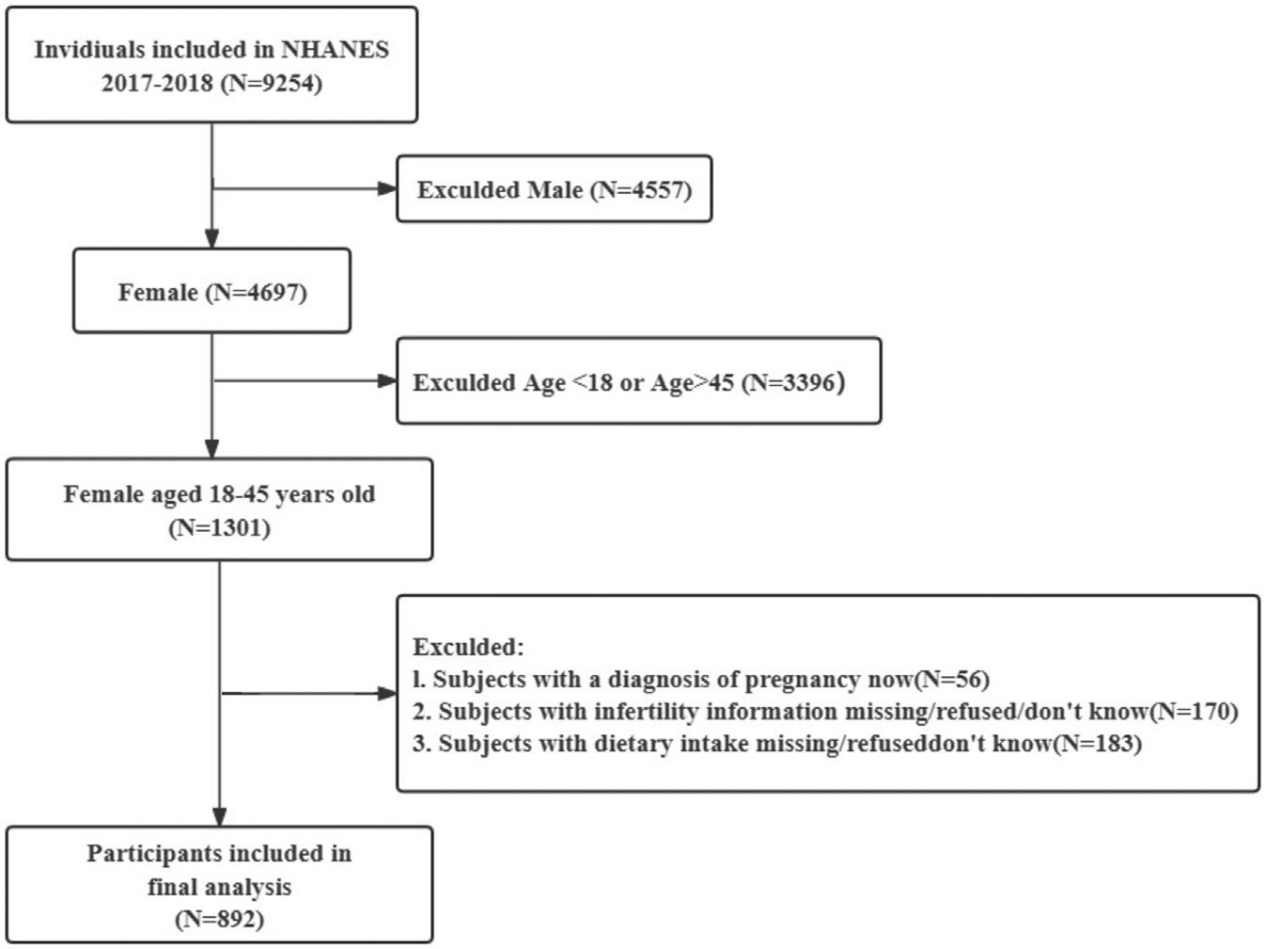
Flow chart of selected participants in this study.

A secondary analysis was performed using the NHANES data (2017–2018). Initially, 9254 non‐institutionalized civilians were included. The inclusion criteria for the present study were the following: (a) female individuals; (b) age 18–45 years, (c) without a diagnosis of pregnancy; (d) with a response to the infertility question; and (e) with and (f) completed two 24‐h diet recalls of DI‐GM components and flavonoid intake. Exclusionary factors included male sex (*n* = 4557), women aged < 18 or > 45 (*n* = 3396), current diagnosis of pregnancy (*n* = 56), females who did not respond to the infertility question (*n* = 170), and incomplete or unreliable two 24‐h dietary recalls of DI‐GM components and flavonoid intake values (*n* = 183) (Figure 1).

### 2.2 Dependent Variables

Dietary data collection in NHANES was conducted through two 24‐h dietary recalls. The first dietary interview was performed in‐person at the NHANES mobile examination center (MEC), followed by a second telephone interview 3–10 days later. The dietary intake values were calculated by averaging data from both recalls, and participants with only one reliable dietary recall were excluded from the analysis.

#### 2.2.1 Dietary Index for Gut Microbiota (DI‐GM)

Dietary index for gut microbiota was developed based on 14 dietary components that were classified into beneficial and unfavorable categories according to their effects on gut microbiota composition. The beneficial components included avocado, broccoli, chickpeas, coffee, cranberries, fermented dairy products, dietary fiber, soybean, whole grains, and green tea. The unfavorable components consisted of refined grains, processed meat, red meat, and a high‐fat diet (defined as ≥ 40% of total energy from fat) (Kase et al. 2024). For the calculation of DI‐GM scores, sex‐specific median intake values were established as threshold criteria for all components, with the exception of high‐fat diet assessment, where a fixed cutoff of 40% energy from fat was applied. Participants were assigned a score of 1 if their consumption exceeded the sex‐specific median for beneficial components or fell below the median for unfavorable components. Conversely, a score of 0 was allocated when intake was below the median for beneficial components or above the median for unfavorable components. The cumulative DI‐GM score, ranging from 0 to 14, was computed by summing individual component scores, with higher values indicating a healthier gut microbiota (Kase et al. 2024).

#### 2.2.2 Dietary Flavonoid Intake

We obtained flavonoid intake data from the USDA Food and Nutrient Database for Dietary Studies (FNDDS) and the NHANES. The present study focused on six subclasses of flavonoids: (1) Anthocyanidins (cyanidin, delphinidin, malvidin, pelargonidin, peonidin, petunidin); (2) flavan‐3‐ols ((−)‐epicatechin*, (−)‐epicatechin 3‐gallate*, (−)‐epigallocatechin*, (−)‐epigallocatechin 3‐gallate*, (+)‐catechin*, (+)‐gallocatechin*, theaflavin, theaflavin‐3,3′‐digallate, theaflavin‐3′‐gallate, theaflavin‐3‐gallate, thearubigins); (3) flavanones (eriodictyol, hesperetin, naringenin); (4) flavones (apigenin, luteolin); (5) flavonols (isorhamnetin, kaempferol, myricetin, quercetin); and (6) isoflavones (daidzein, genistein, glycitein). The sum of all subclasses was considered as total flavonoids (Liu et al. 2023).

We divided the DI‐GM and flavonoid intake into four categories based on the 25th, 50th, and 75th percentiles.

### 2.3 Independent Variables

Self‐reported female infertility was evaluated using the following question: “Have you ever attempted to become pregnant for at least a year without becoming pregnant?” Respondents who answered “yes” were considered female infertile (Liang and Liu 2022).

### 2.4 Covariate Variables

Potential covariate variables based on the existing literature on dietary intake and female infertility were also collected. The demographic variables included age in years, race (“Non‐Hispanic White,” “Non‐Hispanic Black,” “Hispanic,” and “Other”), education (“less than high school degree,” “complete high school degree,” or “higher than high school degree”), marital status (“never married,” “married or living with partner,” or “widowed, divorced, or separated”), and the poverty (categorized into tertiles: < 1.3, 1.3–3.5, and > 3.5) (Yang et al. 2024). Smoking status was defined as never smokers (fewer than 100 cigarettes smoked over a lifetime), former smokers (over 100 cigarettes smoked in the past but no longer smoking), and current smokers (over 100 cigarettes smoked in a lifetime and currently smoking either some days or daily). Alcohol consumption status was categorized into three categories: lifetime abstainers (those who reported drinking alcohol fewer than 12 times in their life), former drinkers (those who drank more than 12 times but have not consumed alcohol in the past year), and current drinkers (those who admit current consumed alcohol more than three times a week) (Li et al. 2023). Participants reported having ever told doctors that they had trouble sleeping (yes/no), or depression according to the 9‐item patient health questionnaire (PHQ) total score (yes: ≥ 10, no: < 10). Furthermore, we investigated other health factors related to fertility, including a history of pregnancy (yes/no), having ever taken birth control pills (yes/no), female hormones taken (yes/no), and pelvic infection (yes/no). Physical activity was divided into two levels: low (< 600 metabolic equivalent minutes [MET‐minutes] per week) and high (≥ 600 MET‐minutes per week) (Guo et al. 2024).

### 2.5 Statistical Analysis

All analyses were conducted applying R version 4.2.1, SAS 9.4 (SAS Institute Inc., Cary, NC, USA) and GraphPad Prism 10. For normally distributed continuous variables, data are presented as mean ± standard deviation (SD) and analyzed using *t*‐tests; for non‐normally distributed data, the median along with the 25th (P25) and 75th (P75) percentiles were compared using Wilcoxon rank‐sum tests. Categorical variables are expressed as frequencies and percentages. According to NHANES weighting guidelines, sampling weights were used to generate a nationally representative estimate. With a weighted univariable logistic model, odds ratios (ORs) and 95% confidence intervals (CIs) were conducted to evaluate the relationships between female infertility and the DI‐GM and flavonoid intake, with the lowest quartile considered as the reference; to evaluate the independent relationships, we used weighted multivariable logistic regression models by controlling for the covariates of age, race/ethnicity, education, BMI, marital status, having ever told a doctor had trouble sleeping, smoking status, alcohol consumption, history of pregnancy, having ever taken birth control pills, female hormones taken, pelvic infection, and physical activity (Wang, Dong, et al. 2024). Finally, to investigate the impact of age on the relationships between DI‐GM, flavonoid intake, and the risk of female infertility, participants were categorized into two groups: < 35 years and ≥ 35 years. Age‐stratified analyses were conducted to examine potential differences in the associations across these two age groups.

**FIGURE 2:**
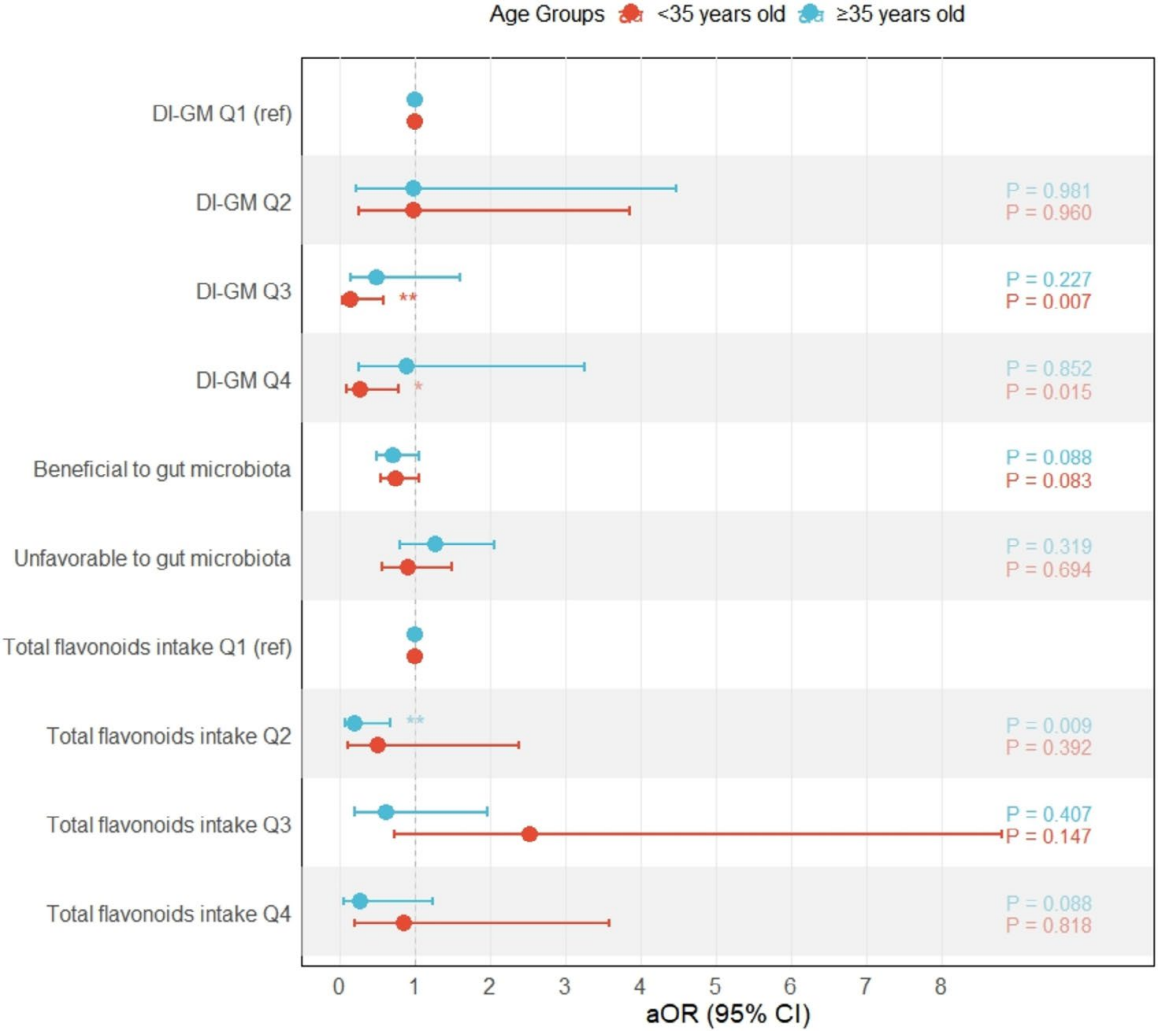
Age‐stratified associations between dietary index for gut microbiota (DI‐GM), total flavonoids intake, and female infertility. Models were adjusted for age, race/ethnicity, education, BMI, marital status, having ever been told by a doctor that they had trouble sleeping, smoking status, alcohol consumption, history of pregnancy, having ever taken birth control pills, female hormones taken, pelvic infection, and physical activity. DI‐GM was additionally adjusted for total flavonoids intake, and total flavonoids intake was additionally adjusted for DI‐GM. *p < 0.05, **p < 0.01.

**FIGURE 3:**
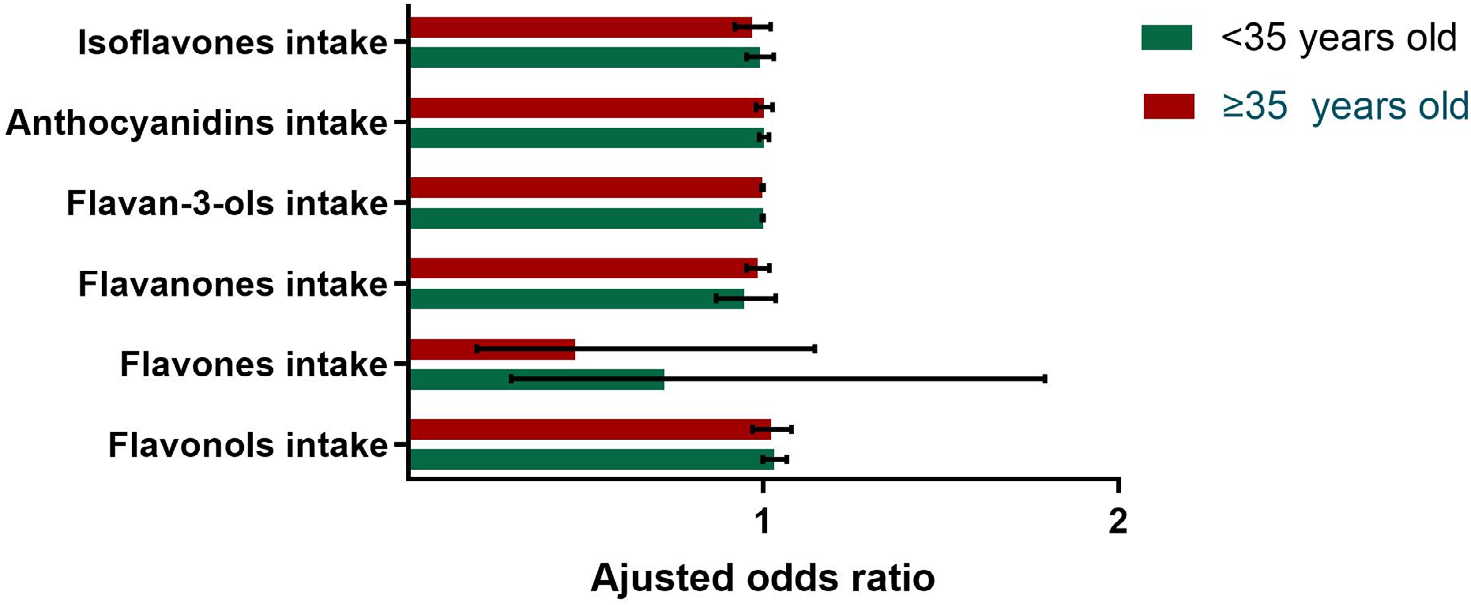
Age‐stratified associations between six flavonoid subclass intakes and female infertility. Models were adjusted for age, race/ethnicity, education, BMI, marital status, having ever told a doctor about having trouble sleeping, smoking status, alcohol consumption, history of pregnancy, having ever taken birth control pills, female hormones taken, pelvic infection, and physical activity, DI‐GM plus the intake of the other five flavonoid subclasses.

Statistical significance was defined as a *p*‐value < 0.05 (Figures 2 and 3).

## 3 Results

### 3.1 Participants Characteristics

Table 1 displays the basic characteristics of the participants with female infertility. This study included 892 females aged 18–45. The prevalence of infertility among the women was 9.4%. The median age was 32 years (IQR: 24–39), with 40.2% of individuals aged ≥ 35 years, and 24.4% were non‐Hispanic White. Overall, 43.8% of participants were obese (BMI ≥ 30), and 22.3% of participants had a poverty ratio of more than 3.5. Most participants (61.7%) had a higher than high school degree, and most (57.5%) were married or living with a partner. Furthermore, 15.4% of the participants were current smokers, and 25.5% had heavy alcohol consumption. Among participants, 25.6% reported telling doctors that they had trouble sleeping, and 11.0% suffered from depression. About 73.6% of the women reported a pregnancy history, 64.6% had taken birth control pills, 5.2% had taken female hormones, and 5.9% reported a pelvic infection. Female participants with an older age, a higher BMI, married or living with a partner, current smoking status, alcohol consumption, having ever told doctors they had trouble sleeping, and having a history of pregnancy were more likely to suffer from infertility (*p* < 0.05).

**TABLE 1:** Characteristics of the US population aged 18–45 years, NHANES, 2017–2018 (*N* = 892).

Demographic characteristics	Total, *n* (%)	Female Infertility		*p* ^a^
		No, *n* (%)	Yes, *n* (%)	
Total	892	808 (90.6)	84 (9.4)	
Age, years	32 (24, 39)	32 (24, 39)	35 (29.5, 39)	0.003*
Age group				
< 35	533 (59.8)	493 (61.0)	40 (47.6)	0.017*
≥ 35	359 (40.2)	315 (39.0)	44 (52.4)	
BMI, kg/m^2^				
< 18.5	32 (3.6)	26 (3.2)	6 (7.1)	0.007*
18.5–24.9	264 (29.6)	248 (30.7)	16 (19.1)	
25–29.9	205 (23.0)	191 (23.6)	14 (16.7)	
≥ 30	391 (43.8)	343 (42.5)	48 (57.1)	
Race/ethnicity				
Non‐Hispanic White	218 (24.4)	199 (24.6)	19 (22.6)	0.285
Non‐Hispanic Black	290 (32.5)	255 (31.6)	35 (41.7)	
Hispanic	217 (24.3)	201 (24.9)	16 (19.0)	
Other	167 (18.7)	153 (18.9)	14 (16.7)	
Poverty				
< 1.3	381 (42.7)	347 (42.9)	34 (40.5)	0.900
1.3–3.5	312 (35.0)	281 (34.8)	31 (36.9)	
> 3.5	199 (22.3)	180 (22.3)	19 (22.6)	
Marital status				
Never married	246 (31.1)	233 (32.8)	13 (16.0)	0.009*
Married or living with partner	455 (57.5)	398 (56.1)	57 (70.4)	
Widowed, divorced, or separated	90 (11.4)	79 (11.1)	11 (13.6)	
Missing/don't know/refused	101			
Education				
Less than high school degree	127 (14.2)	116 (14.4)	11 (13.1)	0.943
Complete high school degree	215 (24.1)	194 (24.0)	21 (25.0)	
More than high school degree	550 (61.7)	498 (61.6)	52 (61.9)	
Smoking status				
Never smoker	650 (72.9)	603 (74.6)	47 (56.0)	0.001*
Past smoker	105 (11.8)	91 (11.3)	14 (16.7)	
Current smoker	137 (15.4)	114 (14.1)	23 (27.4)	
Alcohol consumption				
Never	111 (13.8)	101 (13.8)	10 (13.7)	0.005*
Mild	253 (31.4)	237 (32.4)	16 (21.9)	
Moderate	236 (29.3)	220 (30.1)	16 (21.9)	
Heavy	205 (25.5)	174 (23.8)	31 (42.5)	
Missing/don't know/refused	87			
Had ever told doctor had trouble sleeping				
No	663 (74.4)	611 (75.7)	52 (61.9)	0.006*
Yes	228 (25.6)	196 (24.3)	32 (38.1)	
Missing/don't know/refused	1			
Depression				
No	792 (89.0)	722 (89.6)	70 (83.3)	0.082
Yes	98 (11.0)	84 (10.4)	14 (16.7)	
Missing/don't know/refused	2			
History of pregnancy				
No	209 (26.4)	201 (28.3)	8 (9.9)	0.004*
Yes	582 (73.6)	509 (71.7)	73 (90.1)	
Missing/don't know/refused	101			
Had ever taken birth control pills				
No	316 (35.4)	291 (36.0)	25 (29.8)	0.254
Yes	576 (64.6)	517 (64.0)	59 (70.2)	
Female hormones taken				
No	749 (94.8)	672 (94.8)	77 (95.1)	> 0.999
Yes	41 (5.2)	37 (5.2)	4 (4.9)	
Missing/don't know/refused	102			
Pelvic infection				
No	835 (94.1)	761 (94.7)	74 (89.2)	0.050
Yes	52 (5.9)	43 (5.3)	9 (10.8)	
Missing/don't know/refused	5			
Physical activity, METs min/week				
Low	295 (33.1)	265 (32.8)	30 (35.7)	0.589
High	597 (66.9)	543 (67.2)	54 (64.3)	

^a^ Chi‐square tests and Wilcoxon rank‐sum tests were used to test the differences between the above‐mentioned categories and female infertility.

* *p* < 0.05.

### 3.2 Dietary Intakes of DI‐GM and Flavonoid Intake

Table 2 presents the dietary intakes of flavonoids and DI‐GM scores among participants. The median DI‐GM score for all participants was 5 (IQR: 4, 6). Participants with female infertility had a significantly lower DI‐GM score (3 [IQR: 3, 5.5]) compared to those without female infertility (5 [IQR: 4, 6]; *p* = 0.026). Additionally, the proportions of participants with DI‐GM scores categorized as beneficial or unfavorable to gut microbiota did not differ significantly between participants with and without female infertility (*p* = 0.137 and *p* = 0.202, separately).

**TABLE 2:** Characteristics of DI‐GM and dietary flavonoid intakes among US women aged 18–45 years, NHANES, 2017–2018 (*N* = 892).

Variable	Total	Female Infertility		
		No	Yes	*p*
DI‐GM	5 (4, 6)	5 (4, 6)	3 (3, 5.5)	0.026*
Beneficial to gut microbiota	2 (1, 3)	2 (1, 3)	2 (1, 3)	0.137
Unfavorable to gut microbiota	3 (2, 3)	3 (2, 3)	2 (1.5, 3)	0.202
Total flavonoids intake (mg/day)	58.15 (20.78, 173.29)	59.79 (21.43, 177.70)	49.60 (16.21, 100.04)	0.045*
Isoflavones intake (mg/day)	0.01 (0, 0.18)	0.01 (0, 0.19)	0.01 (0, 0.13)	0.464
Anthocyanidins intake (mg/day)	1.53 (0.03, 11.70)	1.53 (0.04, 12.55)	1.51 (0, 9.11)	0.344
Flavan‐3‐ols intake (mg/day)	15.36 (4.33, 110.67)	15.97 (4.42, 123.97)	11.11 (2.27, 41.47)	0.047*
Flavanones intake (mg/day)	0.21 (0.01, 5.07)	0.22 (0.01, 5.95)	0.09 (0, 1.43)	0.036*
Flavones intake (mg/day)	0.44 (0.13, 1.09)	0.45 (0.14, 1.11)	0.37 (0.11, 0.85)	0.079
Flavonols intake (mg/day)	10.90 (6.00, 18.91)	10.89 (6.06, 18.95)	11.71 (5.06, 18.09)	0.960

Abbreviation: DI‐GM, dietary index for gut microbiota.

* *p* < 0.05.

The total flavonoid intake was significantly lower among participants with female infertility (49.60 mg/day [IQR: 16.21, 100.04]) compared to those without female infertility (59.79 mg/day [IQR: 21.43, 177.70]; *p* = 0.045). Among flavonoid subtypes, flavan‐3‐ols intake was significantly lower in participants with female infertility (11.11 mg/day [IQR: 2.27, 41.47]) compared to those without infertility (15.97 mg/day [IQR: 4.42, 123.97]; *p* = 0.047). Similarly, flavanones intake was lower in participants with female infertility (0.09 mg/day [IQR: 0, 1.43]) compared to those without female infertility (0.22 mg/day [IQR: 0.01, 5.95]; *p* = 0.036). While for other flavonoid subtypes (isoflavones, anthocyanidins, flavones and flavonols), the intake levels showed no statistically significant differences between participants with and without female infertility (*p* > 0.05).

### 3.3 Associations Between DI‐GM, Flavonoid Intake, and Female Infertility

Table 3 presents the associations between DI‐GM, flavonoid intake, and female infertility. In the analysis of DI‐GM scores and female infertility risk, using the lowest quartile (Q1) as the reference group, a significant inverse association was observed in the third quartile (Q3) in both crude (OR = 0.37, 95% CI: 0.15–0.87, *p* = 0.023) and adjusted models (aOR = 0.30, 95% CI: 0.13–0.71, *p* = 0.006). The second quartile (Q2) showed no significant associations in either crude (OR = 0.88, 95% CI: 0.38–2.05, *p* = 0.765) or adjusted models (aOR = 0.85, 95% CI: 0.34–2.15, *p* = 0.735). Similarly, participants in the highest quartile (Q4) demonstrated no statistically significant associations in either the unadjusted model (OR = 0.49, 95% CI: 0.22–1.13, *p* = 0.093) or after adjustment for potential confounders (aOR = 0.49, 95% CI: 0.20–1.20, *p* = 0.120).

**TABLE 3:** Associations between DI‐GM and dietary flavonoid intakes and female infertility among the US women aged 18–45 years, NHANES, 2017–2018 (*N* = 892).

Variable	Model 1^a^		Model 2^b^	
	cOR (95% CI)	*p*	aOR (95% CI)	*p*
DI‐GM				
Q1 (0–3)	1		1	
Q2 (4)	0.88 (0.38–2.05)	0.765	0.85 (0.34–2.15)	0.735
Q3 (5)	0.37 (0.15–0.87)	0.023*	0.30 (0.13–0.71)	0.006*
Q4 (≥ 6)	0.49 (0.22–1.13)	0.093	0.49 (0.20–1.20)	0.120
Beneficial to gut microbiota	0.79 (0.64–0.98)	0.035*	0.75 (0.57–0.98)	0.036*
Unfavorable to gut microbiota	0.96 (0.69–1.33)	0.801	1.06 (0.76–1.50)	0.722
Total flavonoids intake (mg/day)				
Q1 (< 20.78)	1		1	
Q2 (20.78–58.15)	0.45 (0.20–0.98)	0.045*	0.41 (0.17–0.99)	0.047*
Q3 (58.15–173.29)	0.99 (0.46–2.17)	0.986	1.21 (0.53–2.77)	0.645
Q4 (≥ 173.29)	0.35 (0.14–0.91)	0.031*	0.43 (0.16–1.17)	0.099

Abbreviations: 95% CI, 95% confidence interval; aOR, adjusted odds ratio; cOR, crude odds ratio; DI‐GM, dietary index for gut microbiota.

^a^ Model 1: Non‐adjusted model, adjusted for none.

^b^ Model 2: Adjusted for age, race/ethnicity, education, BMI, marital status, had ever told doctor they had trouble sleeping, smoking status, alcohol consumption, history of pregnancy, had ever taken birth control pills, female hormones taken, pelvic infection, and physical activity. Total flavonoids intake (for the DI‐GM model), DI‐GM (for the total flavonoids intake model).

* *p* < 0.05.

When the association between total flavonoid intake and female infertility was analyzed with the flavonoid intake level in quartiles, women in the Q2 showed significantly lower odds of female infertility compared to those in the lowest quartile in the crude model (OR, 0.45; 95% CI, 0.20–0.98; *p* = 0.045). After controlling for the confounders, this inverse association remained statistically significant (aOR, 0.41; 95% CI, 0.17–0.99; *p* = 0.047). In the third quartile (Q3), women showed no statistically significant association in either the crude model (OR: 0.99, 95% CI: 0.46–2.17, *p* = 0.986) or the adjusted model (aOR: 1.21, 95% CI: 0.53–2.77, *p* = 0.645). For the highest quartile (Q4), a significant inverse association was found in the crude model (OR: 0.35, 95% CI: 0.14–0.91, *p* = 0.031); however, this association lost statistical significance after adjusting for confounders (aOR: 0.43, 95% CI: 0.16–1.17, *p* = 0.099). Furthermore, none of the six flavonoid subclasses (isoflavones, anthocyanidins, flavan‐3‐ols, flavanones, flavones, and flavonols) showed statistically significant associations with female infertility in either the crude model or the adjusted model (Table 4).

When analyzing the association between gut microbiota scores and female infertility, beneficial gut microbiota scores presented a significant protective effect with female infertility risk in the fully adjusted model (aOR, 0.75; 95% CI, 0.57–0.98; *p* = 0.036). The association between unfavorable gut microbiota scores and female infertility risk did not reach statistical significance (aOR, 1.06; 95% CI, 0.76–1.50; *p* = 0.722).

These findings suggest that moderate DI‐GM scores (Q3), moderate flavonoid intake (Q2), and beneficial gut microbiota scores are inversely associated with female infertility risk.

**TABLE 4:** Associations between the dietary intakes of six flavonoid subclasses and female infertility among US women aged 18–45 years, NHANES, 2017–2018 (*N* = 892).

Variable	Model 1^a^		Model 2^b^	
	cOR (95% CI)	*p*	aOR (95% CI)	*p*
Isoflavones intake (mg/day)				
Q1 (0.00–0.00)	1		1	
Q2 (0.00–0.01)	0.27 (0.06–1.23)	0.091	0.27 (0.05–1.36)	0.112
Q3 (0.01–0.18)	0.79 (0.39–1.61)	0.514	0.94 (0.42–2.12)	0.876
Q4 (≥ 0.18)	0.64 (0.28–1.47)	0.289	0.78 (0.34–1.78)	0.555
Anthocyanidins intake (mg/day)				
Q1 (0.00–0.03)	1		1	
Q2 (0.03–1.53)	0.63 (0.27–1.50)	0.296	0.69 (0.26–1.88)	0.470
Q3 (1.53–11.70)	1.00 (0.46–2.18)	0.996	1.19 (0.47–3.05)	0.714
Q4 (≥ 11.70)	0.62 (0.26–1.49)	0.284	1.06 (0.38–2.99)	0.912
Flavan‐3‐ols intake (mg/day)				
Q1 (0.00–4.33)	1		1	
Q2 (4.33–15.36)	0.92 (0.41–2.03)	0.826	0.83 (0.32–2.17)	0.702
Q3 (15.36–110.67)	1.02 (0.46–2.26)	0.962	1.33 (0.56–3.19)	0.520
Q4 (≥ 110.67)	0.66 (0.27–1.64)	0.374	0.42 (0.15–1.24)	0.117
Flavanones intake (mg/day)				
Q1 (0.00–0.03)	1		1	
Q2 (0.03–1.53)	0.88 (0.40–1.96)	0.756	1.27 (0.55–2.92)	0.577
Q3 (1.53–11.70)	0.51 (0.22–1.19)	0.120	0.76 (0.29–2.01)	0.575
Q4 (≥ 11.70)	0.44 (0.18–1.09)	0.077	0.44 (0.15–1.35)	0.152
Flavones intake (mg/day)				
Q1 (0.00–0.01)	1		1	
Q2 (0.01–0.21)	0.94 (0.42–2.09)	0.870	0.99 (0.43–2.30)	0.982
Q3 (0.21–5.07)	0.49 (0.22–1.11)	0.089	0.59 (0.23–1.52)	0.274
Q4 (≥ 5.07)	0.45 (0.18–1.13)	0.090	0.41 (0.14–1.20)	0.103
Flavonols intake (mg/day)				
Q1 (0.00–6.00)	1		1	
Q2 (6.00–10.90)	0.92 (0.38–2.20)	0.845	0.95 (0.34–2.63)	0.913
Q3 (10.90–18.91)	1.02 (0.46–2.27)	0.953	1.95 (0.66–5.70)	0.225
Q4 (≥ 18.91)	0.76 (0.31–1.88)	0.554	2.33 (0.67–8.19)	0.186

Abbreviations: 95% CI, 95% confidence interval; aOR, adjusted odds ratio; cOR, crude odds ratio.

^a^ Model 1: Non‐adjusted model, adjusted for none.

^b^ Model 2: Adjusted for age, race/ethnicity, education, BMI, marital status, had ever told doctor they had trouble sleeping, smoking status, alcohol consumption, history of pregnancy, had ever taken birth control pills, female hormones taken, pelvic infection, and physical activity, DI‐GM plus the intake of other 5 flavonoid subclasses.

### 3.4 Age‐Stratified Analysis of DI‐GM, Flavonoid Intake, and Female Infertility

When stratified by age (< 35 years and ≥ 35 years), different patterns emerged in the associations between DI‐GM, flavonoid intake, and female infertility risk (Figure 2). In women aged < 35 years, DI‐GM Q3 showed a protective effect against female infertility (aOR = 0.13, 95% CI: 0.03–0.58, *p* = 0.007) compared to Q1. DI‐GM Q4 also showed a significant inverse association with female infertility risk (aOR = 0.27, 95% CI: 0.09–0.77, *p* = 0.015) in this age group. However, for women aged ≥ 35 years, these associations were not statistically significant (Q3: aOR = 0.48, 95% CI: 0.14–1.59, *p* = 0.227; Q4: aOR = 0.88, 95% CI: 0.24–3.25, *p* = 0.852).

When stratified by age, the beneficial gut microbiota scores showed similar trends in both age groups but did not reach statistical significance (< 35 years: aOR = 0.74, 95% CI: 0.53–1.04, *p* = 0.083; ≥ 35 years: aOR = 0.71, 95% CI: 0.48–1.05, *p* = 0.088). The unfavorable gut microbiota scores showed no significant associations in either age group (< 35 years: aOR = 0.91, 95% CI: 0.55–1.49, *p* = 0.694; ≥ 35 years: aOR = 1.27, 95% CI: 0.79–2.05, *p* = 0.319).

For total flavonoid intake, no significant relationships were identified between quartiles and female infertility risk among women under 35 years of age (Q2: aOR = 0.51, 95% CI: 0.11–2.37, *p* = 0.392; Q3: aOR = 2.52, 95% CI: 0.72–8.79, *p* = 0.147; Q4: aOR = 0.84, 95% CI: 0.20–3.58, *p* = 0.818). Among women aged ≥ 35 years, Q2 showed a significant protective effect against female infertility risk (aOR = 0.19, 95% CI: 0.06–0.66, *p* = 0.009), while Q3 (aOR = 0.61, 95% CI: 0.19–1.95, *p* = 0.407) and Q4 (aOR = 0.26, 95% CI: 0.05–1.23, *p* = 0.088) showed no statistically significant associations with female infertility risk.

In the analysis of flavonoid subclasses, none of the six subclasses (isoflavones, anthocyanidins, flavan‐3‐ols, flavanones, flavones, and flavonols) showed statistically significant associations with female infertility risk in either age group (*p* > 0.05) (Figure 3).

This study suggests that the protective effects of DI‐GM and flavonoid intake on female infertility risk may vary by age, with DI‐GM showing significant protective effects at higher (Q3) and highest (Q4) levels in women aged < 35 years, while total flavonoid intake shows significant protective effects in the second quartile among women aged ≥ 35 years. The beneficial gut microbiota scores showed consistent protective associations across age groups, though statistical significance was only observed in the overall population.

## 4 Discussion

Our study investigated the relationships between DI‐GM, dietary flavonoid intake, and female infertility in women aged 18–45 years in the United States, utilizing data from the NHANES 2017–2018 survey. Our findings revealed significant inverse associations between higher DI‐GM scores, moderate flavonoid intake, and female infertility risk, with beneficial gut microbiota scores showing protective effects. Furthermore, our findings revealed age‐specific patterns in the associations between DI‐GM, flavonoid intake, and female infertility risk. Additionally, these associations demonstrated distinct patterns in age‐stratified analyses.

### 4.1 DI‐GM and Female Infertility

Our results demonstrated that higher DI‐GM scores, which reflect healthier gut microbiota, were significantly linked to reduced risk of female infertility. Higher intake of beneficial components including avocado, broccoli, chickpeas, coffee, cranberries, fermented dairy products, dietary fiber, soybean, whole grains, and green tea, contributed to a composite beneficial gut microbiota score that was linked to reduced risk of female infertility. These findings emphasize the potential value of adopting gut microbiota‐friendly dietary patterns to support reproductive health. The biological plausibility of this association is supported by previous research (Beni et al. 2024; Shan et al. 2024), which found that dysbiosis, or an imbalance in gut microbiota, has been implicated in various reproductive disorders that contribute to infertility (Qi et al. 2021). For instance, gut microbiota dysbiosis is associated with PCOS (Sun et al. 2023) and metabolic disorders (Hamjane et al. 2024), which are known risk factors for infertility. Gut microbiota have been shown to influence reproductive health through multiple pathways, including regulation of circulating levels of steroid sex hormones, immune system function, insulin sensitivity, and gonadal microbiota (Qi et al. 2021). Moreover, a healthy gut microbiota may also contribute to reduced female infertility risk through its role in estrogen metabolism regulation (Wang, Sang, et al. 2024) and systemic inflammation control (Liu et al. 2024; Wang, Zheng, et al. 2024). Our findings suggest that dietary interventions incorporating DI‐GM screening and improving beneficial gut microbiota scores through increased consumption of beneficial dietary components (involving avocado, broccoli, chickpeas, coffee, cranberries, fermented dairy products, dietary fiber, soybean, whole grains, and green tea) may represent a promising strategy for female fertility management. Further age‐stratified analyses revealed more robust protective effects among women aged < 35 years, where both moderate (Q3) and highest (Q4) DI‐GM scores showed significant protective associations. However, these protective effects were not observed in women aged ≥ 35 years, suggesting that the influence of healthier gut microbiota dietary patterns on female fertility might be more pronounced during earlier reproductive years. This age‐specific pattern highlights the potential importance of early dietary intervention targeting gut microbiota for optimal reproductive health outcomes.

### 4.2 Dietary Flavonoid Intake and Female Infertility

Our research also found that, after adjusting for potential confounders, moderate total flavonoid intake was linked to a reduced risk of female infertility. This finding supports growing evidence from recent studies demonstrating the beneficial effects of flavonoids on reproductive health (Sirotkin and Harrath 2024; Zhang et al. 2022). For instance, evidence demonstrated that flavonoids may contribute to female fertility outcomes through several pathways, such as promoting the development of follicles in ovaries, supporting embryonic and ovarian cell survival and growth, enhancing fertility, and regulating the synthesis of reproductive hormones (Sirotkin and Harrath 2024). Interestingly, while total flavonoid intake showed protective effects, individual flavonoid subclasses did not demonstrate significant associations, highlighting the importance of considering the combined effects of different flavonoids rather than individual compounds. This discrepancy might be attributed to the relatively small sample size, which limited statistical power for subclass analyses, or it may reflect the complex interactions among flavonoid compounds rather than the effects of individual components (Hajimehdipoor et al. 2014; Hidalgo et al. 2010). This evidence highlights the importance of considering the combined effects of flavonoids rather than focusing solely on individual subclasses. To further understand the observed protective effects of flavonoids, it is essential to explore the underlying biological mechanisms. The protective effects of flavonoids against female infertility are likely mediated by their well‐documented antioxidant properties. Evidence showed that excessive ROS production has been shown to compromise ovarian function, leading to detrimental effects on both follicular development and oocyte quality (Sirotkin and Harrath 2024), which are critical determinants of female fertility. When the body's natural antioxidant defense mechanisms are overwhelmed by excessive ROS, oxidative stress ensues, potentially resulting in unexplained infertility (Al‐Gubory et al. 2010). Therefore, the biological plausibility of our findings is supported by the capacity of flavonoids to act as potent antioxidants, effectively mitigating oxidative stress, reducing ROS levels, and protecting ovarian function. These mechanisms may collectively explain the observed association between moderate flavonoid intake and reduced female infertility risk.

Moreover, age‐stratified analyses revealed distinct patterns in the association between flavonoid intake and female fertility: a protective effect was observed specifically among women aged ≥ 35 years, while no significant associations were found in women aged < 35 years (Ra et al. 2023). This age‐specific pattern aligns with our current understanding of oxidative stress in reproductive aging.

First, oxidative stress naturally increases with age, particularly affecting reproductive tissues. The ovaries are especially susceptible to age‐related oxidative damage, which can compromise oocyte quality and overall reproductive function (Shang et al. 2024). Studies have shown that aging ovaries exhibit elevated levels of oxidative stress markers and reduced antioxidant capacity (Shang et al. 2024). Second, while physiological levels of ROS are essential for normal reproductive processes, including ovulation, excessive oxidative stress can be detrimental (Shang et al. 2024). As women age, maintaining this delicate balance becomes more challenging, as evidenced by the increased oxidative damage observed in aging reproductive tissue s (Ra et al. 2023; Shang et al. 2024).

Therefore, the stronger protective effect of flavonoids observed in women aged ≥ 35 years may reflect their greater need for antioxidant support to counteract age‐related increases in oxidative stress (Ra et al. 2023). In contrast, younger women might have more robust endogenous antioxidant systems capable of maintaining optimal ROS levels without additional dietary support. Our study indicates that flavonoids may play an important role in supporting female reproductive health, primarily due to their antioxidant and anti‐inflammatory effects. The present study provides a mechanistic basis for the potential application of flavonoids in the prevention and management of female infertility. Furthermore, dietary strategies that include flavonoid‐rich foods represent a simple, cost‐effective strategy to support reproductive health. However, the precise mechanisms underlying these age‐specific associations require further investigation through experimental studies. Future research should explore the potential molecular pathways involved and evaluate the clinical efficacy of flavonoid supplementation in improving female fertility outcomes.

This study has several limitations. First, the cross‐sectional design allows us to identify associations between DI‐GM, flavonoid intake, and female infertility, but does not establish causation. These associations need to be further demonstrated through prospective cohort research and well‐designed RCTs. Second, only female infertility was investigated; therefore, our results may not apply to male infertility. Third, the relatively small sample size may have reduced statistical power, suggesting the need for larger‐scale studies to validate these findings. Additionally, the age‐specific patterns observed in our study warrant further investigation in larger cohorts to better understand the underlying mechanisms.

Despite these limitations, this study provides novel epidemiological evidence for the associations of DI‐GM and flavonoid intake with female infertility in US women of reproductive age. Our findings suggest that:

1. Screening and monitoring: Implementation of DI‐GM screening and regular assessment of total flavonoid consumption should be considered in reproductive‐aged women, especially those with infertility risk factors, to identify potential dietary intervention needs and improve fertility outcomes.
2. Personalized dietary counseling: Early dietary counseling focusing on gut microbiota‐friendly foods (e.g., avocado, broccoli, chickpeas, coffee, cranberries, fermented dairy products, dietary fiber, soybean, whole grains, and green tea) and achieving appropriate flavonoid intake levels should be initiated before conception is contemplated, as these dietary patterns may promote a healthier gut microbiota and reduce oxidative stress, thereby contributing to better reproductive health.
3. Tailored interventions for different life stages: Women under 35 years of age may benefit more from dietary interventions aimed at improving gut microbiota composition, while women aged 35 years and older may require additional strategies, including appropriate flavonoid dietary intake, to address age‐related oxidative stress.

In conclusion, our study provides novel epidemiological evidence that higher DI‐GM scores and moderate flavonoid intake are linked to a reduced risk of female infertility among US women of reproductive age. Age‐specific patterns were observed, with higher DI‐GM dietary patterns demonstrating significant protective effects in younger women (< 35 years), while flavonoid intake showed significant protective effects in women aged 35 years and above. These findings highlight the importance of personalized dietary strategies that address the unique reproductive health needs at different life stages. This study highlights the potential of dietary interventions targeting gut microbiota composition and flavonoid intake as simple, cost‐effective strategies for female infertility prevention and management. Further investigation of these associations may inform the development of evidence‐based dietary strategies for reproductive health optimization.

## Author Contributions

**Di Xiao:** formal analysis (equal), writing – original draft (equal), writing – review and editing (equal). **Xiang Sun:** methodology (equal), writing – review and editing (equal). **Weidong Li:** data curation (equal). **Zihao Wen:** data curation (equal). **Wei‐Hong Zhang:** supervision (equal), writing – review and editing (equal). **Li Yang:** conceptualization (equal), writing – review and editing (equal).

## Ethics Statement

The NHANES protocol was sanctioned by the Ethics Review Board of the National Centers for Health Statistics. Informed consent was obtained from all individual participants included in the study.

## Conflicts of Interest

The authors declare no conflicts of interest.

## Data Availability

Data of this study can be accessed on the official website of NHANES (https://wwwn.cdc.gov/nchs/nhanes/default.aspx).
